# Municipalities and Public Health

**Published:** 1891-08-22

**Authors:** 


### The Hospital

A Weekly Journal of

**Science, Medicine, Nursing, and Philanthropy.**

*For Hospitals, Asylums, Infirmaries, Dispensaries, Medical Practitioners, Students, Nurses, and the Charitable Public.*

Vol. X.—No. 256.] [Aug. 22, 1891.

### MUNICIPALITIES AND PUBLIC HEALTH.

Dr. Billings, the eminent American physician, whose writings are famous all the world over, has done good public service by the preparation of his address on public health and municipal government, which was delivered to the members of the American Academy of Political and Social Science. In this country municipalities have shown enormous development in matters of Public Health. The conditions of the people question has become of late years the question of questions in most cities and boroughs. Birmingham and Glasgow are two notable examples of the advantages which a population can secure to itself, without undue taxation, by selecting for local offices representatives who possess commendable pride and take an intelligent interest in, all that concerns the welfare of their own locality and its inhabitants. Parks, free libraries, baths and wash-houses, steam tramways, art galleries, the opening of museums on Sundays, the enforcement of local bye-laws repressing nuisances and securing the peaceful enjoyment of the Sabbath for all classes, may be enumerated amongst the various advantages which the best governed of our towns have now secured for themselves to the benefit of everybody. If we add to these a pure and abundant supply of water and gas, and a prospect of the early freeing of the smaller tenement houses from rates, with the provision of a separate tenement or house for each poor family at a rental which shall not exceed one-eighth of the weekly wages of the labouring class; surely, all who are interested in municipal institutions may thank God and take courage. No doubt much more might be done, and undoubtedly will be done, because the people as they become better educated take a more intelligent interest in everything, and so demand opportunities for improving their material and mental condition to the no small advantage of the nation. The free institutions of this country have at times led to outbreaks of sentimentalism, which have been justly characterised by foreigners as ludicrous if not contemptible. Still, when all is said that can be said by way of criticism, the fact remains that the people who reside in our large provincial cities now possess advantages which have never been exceeded by any citizens in the history of the world.

Dr. Billings shows that in America, as in England, there has been a rapid increase in the population of the cities, due mainly to immigrants from foreign countries, and to migration from rural districts to the cities. This migration from rural districts is an increasing feature in American life, and threatens to have equally severe effects upon agricultural communities in that country to those it is likely to have in England to-day. Dr. Billings next shows that the aggregation of large bodies of men in limited localities, though it has many advantages (because it concentrates force, stimulates and makes possible the doing of many things conducive to human happiness and progress, which would not be undertaken in a scattered community), still is attended with serious dangers to health. There is much cause for thankfulness, however, in the fact that the average duration of life of the city dwellers is now fifty per cent. more than and the death rate one hundred per cent. less in the healthiest towns than it was formerly. Of course the young, the ambitious, the energetic, the best of the race, regard health not as an end but as a means, and so they flock into cities still as they formerly flocked, regardless of the risks, because there they have reasonable hope of procuring pleasures, prizes, and rewards, possibly at the risk of their lives, but otherwise unattainable.

Isolation and solitude to a man of enterprise and ability are intolerable hindrances to progress which render life not only unenjoyable but unendurable. Hence the rush to the cities, and from the smaller cities to the larger cities, or, at any rate, so far as the best minds are concerned, to those centres of population where there are intellectual life and continuous progress. Dr. Billings makes a good point by proving that where the trade pulse beats strongly and clearly, there the inhabitants are indifferent to health problems, and it is hard to persuade such a city that it is ill, or in danger of becoming ill, so long as business prosperity attends the labours of its citizens. Dr. Billings regards this as a healthy sign, because he holds that cities, like individuals, may be too pessimistic in regard to health questions, and quotes Paget in support of this view, as follows:— "He should not be deemed thoroughly healthy who is made better or worse, more or less unfit for work, by every change of weather or food, nor he who, in order that he may do his work, is bound by exact rules of living." The health of a community, like that of a man, is a "function of many variables;" like causes under like circumstances will produce like effects in the microcosm as well and as certainly as they will in the test-tube; but one may have like effects from different combinations of causes. We have no space to follow Dr. Billings in the elaboration of this view. He shows by a reference to the death-rate of a particular ward or block and the occupation of foul rooms, the character of the individuals who inhabit them. The separation of the evil from the good, and the healthy from the sick; the duty which one man owes to his neighbour, and the wisdom of attending to this divine rule; the importance of a separate habitation for each family, not only on health grounds, but as proving the greater intelligence and industry of the people, since the demand creates the supply; the comparison of death-rates and sick-rates, and overcrowding, are all matters of grave consideration which find a place in Dr. Billings' address.

In all these matters American cities seem to be behind our own, and Dr. Billings reluctantly quotes this statement by Mr. Andrew D. White: "Without the slightest exaggeration we may assert that with few exceptions the city Governments in the United States are the worst in Christendom—the most expensive, the most inefficient, and the most corrupt." It is well sometimes to see ourselves as others see us, but we may in the face of a statement like this, by so eminent an authority, congratulate ourselves upon the fact that the evils referred to by Mr. White do not find a prominent place, if they find a place at all, in our system of municipal government at the present day. For our own part, having devoted a great deal of time to the personal inspection of the homes of the people in the cities of this and other countries, we shall never rest content until each municipality determines to provide, and does in effect provide, a separate tenement for every family, however poor.

The State now provides County Lunatic Asylums for the poorer classes when mentally afflicted, thus relieving their families of the burden of their maintenance, which used formerly to devolve upon them. Yet this action of the State in no way pauperises or injures those who are benefited by it. Free education is now placed within the reach of every ratepayer; the public at large are protected from the criminal classes by prisons and industrial schools, and there seems no sound objection to going a step further by the adoption of a system on the part of municipalities, which by the destruction and extinction of the slums will remove the nests and nurseries from which the vicious and criminal classes spring. This can be done without increasing existing taxation to any great extent, because the expenditure upon the various agencies which punish and repress vice and crime must necessarily be thereby diminished considerably.

In other words the corporations throughout the country which now control, and own in many instances, the water and gas, and which are responsible for the enforcement of the law and the collection of the rates, might be empowered to supply at their discretion certain of the houses occupied by the poorer classes of the inhabitants with free water and free gas, and to exempt such buildings from the payment of rates. The value of the local rates equals about twenty per cent. of the rent of such property, and the value of the water and gas is about ten per cent. more, making thirty per cent. in all. This represents about the difference between what the poorest can pay, and what the letting value of such property must be to make it a commercial success.

Here then seems a reasonable solution to a difficult problem, but its adoption must be guarded by the corporations undertaking the ownership of this class of houses which should henceforth be managed entirely by their officers, and be wholly under their own control. Unless these properties are under the control of the authorities great abuses would undoubtedly attach to them. The corporations can borrow money cheaper than any one else, say at three per cent., and as these calculations have all been based upon a nett profit of four per cent., they would be enabled to repay the principal sums in a term of years by means of a sinking fund, and to adjust the price of sites out of the profit of one per cent. per annum. The adoption of such a scheme as this must not be made by one town only, but by all well-administered municipalities, or the poor would be attracted into particular towns to their own great suffering and disappointment, and the serious injury of the community.

